# A rare case report of splenic infarction in a previously healthy teenager caused by acute infectious mononucleosis

**DOI:** 10.1097/MD.0000000000039170

**Published:** 2024-08-02

**Authors:** Lijun Ma

**Affiliations:** a Department of Internal Medicine, Tianjin United Family Hospital, Tianjin, China.

**Keywords:** Epstein–Barr virus, infectious mononucleosis, splenic infarction

## Abstract

**Rationale:**

Splenic infarction usually occurs in patients with underlying illnesses such as thromboembolic disorders and infiltrative hematologic diseases. Herein, we report a rare case of splenic infarction in a previously healthy boy diagnosed with infectious mononucleosis (IM). Splenic infarction is a rare complication of IM and its incidence is unknown. This case report summarizes the clinical characteristics, treatment options, and anticipated time for recovery from splenic infarction in IM.

**Patient concern:**

A16-year-old boy presented to our clinic with complaints of fever, sore throat, and general sweakness for 7 days. The patient was diagnosed with IM due to an Epstein–Barr virus infection. Two days later, the patient developed severe abdominal pain in the left upper quadrant and returned to our ER for further evaluation.

**Diagnosis:**

IM complicated with splenic infarction.

**Interventions:**

Contrast-enhanced CT confirmed the diagnosis of splenic infarction. This patient was admitted for supportive treatment and close medical monitoring. Surgical

**Outcomes:**

The patient recovered well with conservative treatment.

**Lessons:**

IM is most often seen in adolescents and young adults. Splenic infarction is a rare complication of IM, particularly in patients who do not usually have any underlying predisposing medical conditions. Contrast-enhanced CT is the imaging modality of choice in suspected cases. Early recognition and treatment of splenic infarction in patients with IM can help prevent potentially life-threatening events. Patients should be advised to avoid sports that may precipitate splenic rupture. However it is still unknown when it is safe for patients to resume sports. In our case, 6 weeks after the splenic infarction, the patient generally felt well with complete resolution of objective symptoms and splenomegaly, and resumed sports without experiencing any adverse events.

## 1. Introduction

Infectious mononucleosis (IM) is a clinical syndrome usually caused by Epstein–Barr virus (EBV) and is characterized by fever, sore throat, lymphadenopathy, and splenomegaly.^[1]^ Fever and lymphadenopathy are present in approximately 90% of the patients with infectious mononucleosis (IM). Most cases of symptomatic IM occur in adolescents and young adults. In most patients, IM is a benign, self-limiting illness that only requires symptomatic treatment. However, IM can also lead to severe life-threatening complications, with splenic rupture being the most reported one.^[2]^ Splenic infarction is a rare complication of IM, especially in individuals without underlying hematologic comorbidities.^[3]^ We present the case of a young healthy male diagnosed with IM due to EBV complicated by splenic infarction.

## 2. Case presentation

A previously healthy 16-year-old boy presented with a chief complaint of fever and worsening sore throat that started 7 days before the visit. The patient had a blood pressure of 132/72 mm Hg, heart rate of 85 beats/min, respiratory rate of 18 breaths/min, and body temperature of 38.9 °C. Physical examination revealed tonsillar exudate and bilateral enlarged tender cervical lymphadenopathy. The patient had regular heart rate and rhythm with no murmurs; the lungs were clear with no rales, wheezing, or abnormal breathing sounds, and the abdomen was soft and non-tender.

Complete blood count (CBC) results were as follows: white blood cells (WBC), 19.6 × 10^9^/L; lymphocytes, 8.1 × 10^9^/L; monocytes, 2.91 × 10^9^/L; red blood cells, 5.5 × 10^12^/L; hemoglobin, 153 g/L; and platelets, 212 × 10^9^/L. A peripheral blood smear revealed elevated monocyte (18%) and lymphocyte (55%), atypical lymphocytes (2%), and decreased neutrophil (25%). The inflammatory markers C-reactive protein (CRP) and procalcitonin (PCT) were elevated to 23 mg/L and 0.19 ng/mL, respectively. The liver enzyme levels were all within normal ranges. The EBV viral capsid antigen (VCA) immunoglobulin M (IgM) test was positive. The patient was diagnosed with IM caused by EBV, and tonsillitis.

The patient received antimicrobial treatment with cefuroxime and symptomatic treatment with oral nonsteroidal anti-inflammatory medications (NSAIDs). The patient was a member of a school football team. The patient was advised not to participate in any athletic activities for at least 4 weeks from the onset of symptoms.

**Figure 1.**
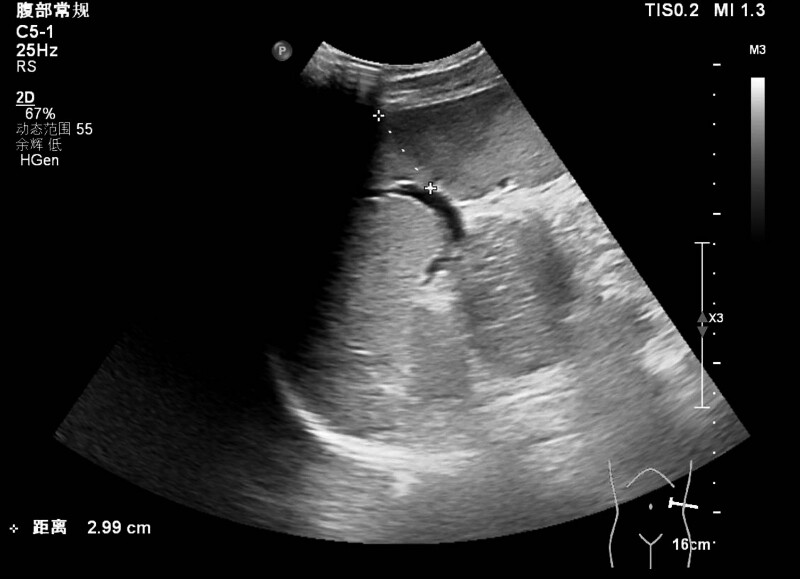
Ultrasound showing a hypoechoic wedge-shaped region of splenic tissue.

**Figure 2.**
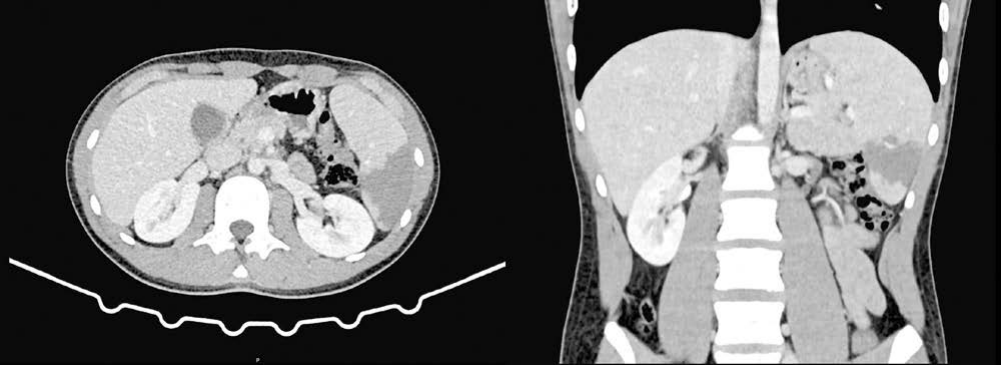
Contrast-enhanced abdominal CT showing a wedge-shaped zone of hypodensity on the spleen tissue.

Two days later (9th day after the onset of symptoms), the patient presented to our emergency department with sudden onset of persistent severe abdominal pain. The patient also complained of nausea but no vomiting. The pain was mainly in the left upper quadrant with tenderness on palpation. The patient denied any traumatic causes and described the pain as persistent intense dull pain that worsened with movement or even deep breathing. Abdominal examination revealed a soft, non-distended abdomen with tenderness in the left quadrant and hyperactive bowel sounds. Abdominal pain may suggest complications involving the spleen in IM patients. Urgent abdominal ultrasonography revealed splenomegaly with an uncertain hypoechoic region (Fig. 1). Therefore, splenic infarction was suspected. Contrast-enhanced abdominal computed tomography (CT) was performed for further investigation. A large wedge-shaped zone of hypodensity on the spleen was found, with the apex pointing towards the helium, which indicated splenic infarction (Fig. 2). Blood tests showed that the WBC (13.3 × 10^9^/L) and CRP (14 mg/L) levels were still elevated but lower than those in previous tests 2 days earlier; liver function, renal function, coagulopathy tests, and D-dimer levels were all within normal limits. The patient was clinically stable but could not tolerate normal oral intake due to abdominal pain and nausea. The patient was admitted for close medical monitoring and supportive treatment. Intravenous fluid was administered to maintain hydration. Ibuprofen and oxycodone were prescribed as needed to manage the pain. Cefuroxime prescribed at the last visit was also continued to treat the pharyngeal infection. The patient was instructed to rest in bed with reduced physical activity as tolerated, to avoid stimulating the infarcted spleen. A comprehensive investigation of the patient’s medical and family histories revealed no remarkable findings. The intensity of the abdominal pain gradually decreased after supportive treatment. The patient was able to resume normal oral intake on the second day of admission. The patient did not exhibit any signs of splenic abscesses, bleeding, rupture, or progressing infraction. The patient was discharged home 3 days later and was recommended to refrain from any vigorous physical activities until further notice.

## 3. Outcomes

Two weeks after splenic infarction, the patient returned for follow-up. The patient has been able to perform all activities of daily living without any symptoms. There were no outstanding findings on physical examination, except for a slightly enlarged non-tender lymph node in the right cervical area. The results of blood tests, including CBC, liver function, renal function, and CRP, were all within normal ranges.

Six weeks after splenic infarction, the patient continued to feel well and requested return to sports. Abdominal ultrasonography was performed and confirmed the resolution of splenomegaly. The patient was advised to resume sports, starting with reduced intensity and increasing gradually as tolerated.

Three months after splenic infarction, during a phone follow-up, the patient reported that he was in good health and did not experience any physical discomfort during sports. The patient recovered well without adverse outcomes.

## 4. Discussion

EBV is the most common cause of IM. EBV, also known as human herpesvirus 4, belongs to the Herpesviridae family and Gammaherpesvirinae subfamily and can modulate a plethora of biological processes in viral-associated cancers. EBV is also associated with several human malignancies, such as lymphoma, nasopharyngeal carcinoma, and gastric cancer.^[4]^ EBV is a prevalent virus that infects more than 90% of the global population. Saliva is the primary route for EBV transmission. EBV directly infects the epithelial cells and B cells in the oral cavity. The primary infection is typically asymptomatic, but 35% to 50% of the human adolescent population develops IM approximately 1 month after the initial infection, and the virus persists throughout an individual’s life.^[5]^ Occasionally, IM can also be caused by a number of other pathogens, such as cytomegalovirus (CMV), herpes simplex virus(HSV), human immunodeficiency virus (HIV), human herpesvirus 6 (HHV-6), and toxoplasma, but the symptoms are often milder than EBV associated IM.

IM is characterized by the main symptoms of a fever, sore throat, cervical or generalized lymphadenopathy and fatigue. Among these symptoms, fatigue and cervical lymphadenopathy typically persist for at least 3 weeks. Splenomegaly occurs in approximately 50% of the IM patients.^[6]^ The common symptoms of IM can lead the treating physician to suspect bacterial pharyngotonsillitis, especially in some atypical cases. A complete blood count can be helpful in distinguishing IM from bacterial pharyngotonsillitis. An absolute lymphocyte count > 4 × 10^9^/ L or ≥ 10% atypical lymphocytes indicate IM. The atypical lymphocytes are activated CD8 + T cells that respond to EBV-infected B cells.^[7]^ Proliferation of EBV-infected B cells and activation of T cells trigger the production of several cytokines that induce rapid spleen enlargement in IM patients. Elevation of liver enzymes can be observed in 75% of the IM patients,^[6]^ and the presence of elevated liver enzymes also raises the clinical suspicion of IM. All patients with suspected IM should undergo EBV-specific antibody testing, and a positive anti-VCA IgM antibody test can be used to diagnose acute EBV infection. In theory, treatment with antiviral agents (acyclovir and valacyclovir) may be effective in preventing viral replication, which helps to keep the virus inactive. However, no high-quality studies have provided evidence to support the use of antiviral agents for IM. Therefore, treatment of IM is mainly supportive, and routine use of antivirals is not recommended. Most patients with IM recover without any major complications, although fever and fatigue may last for weeks.

Spleen infarction is usually caused by impaired blood supply to the spleen due to occlusion of the splenic artery or one of its subbranches. It is a rare cause of abdominal pain and usually occurs in a person with an underlying hematologic disorder, hypercoagulable state, blood-borne malignancy, or embolic illness, such as sickle cell anemia, lymphoma, leukemia, atrial fibrillation, or endocarditis.^[8]^ Yen et al^[9]^ reported that atrial fibrillation is the most common predisposing condition for splenic infarction. IM primarily affects both teenagers and young adults. This group of individuals is usually healthy and does not have any potential chronic medical issues, as mentioned above. Splenic infarction is a rare complication of IM, and its exact incidence is unknown. The pathophysiological mechanisms of splenic infarction in IM are still not well studied, however, in theory, there are several important factors that may contribute to this process. Splenomegaly in IM may place patients at risk for splenic infarction, because the rapidly expanding spleen causes relatively insufficient blood supply to the parenchyma. IM is often associated with a transient hypercoagulable state induced by an inflammatory response to an acute viral infection. Similarly, multiple cases of splenic infarction secondary to COVID-19 infection were reported during the epidemic, which also indicated an association between hypercoagulable states and splenic infarction during viral infection.^[10,11]^ Dehydration due to the illness, combined bacterial infection can also facilitate the development of splenic infarction by promoting hypercoagulability.

The most commonly identified signs and symptoms of splenic infarction are left upper quadrant abdominal pain and tenderness, nausea, and vomiting.^[12]^ Splenic infarction or rupture should be suspected in patients with IM who present with left upper quadrant abdominal pain. Abdominal ultrasonography is readily available in most clinical settings and can be used for initial evaluation. The infarcted area usually appears as a patchy hypoechoic lesion on ultrasonography.^[13]^ However, ultrasonography has a low sensitivity for acute splenic infarction. Abdominal CT performed with intravenous contrast remains the best method to confirm the diagnosis of splenic infarction, especially for patients in the hyperacute phase. The infarcted tissue appears as a wedge-shaped hypoenhancing area, with the apex pointing toward the helium and the base of the splenic capsule.

Supportive care to relieve symptoms is usually sufficient in stable patients with splenic infarction, and most patients recover without specific complications. However, splenic infarction can also lead to, such as abscesses, pseudo-cyst formation, bleeding, or spleen rupture. Hospital admission may be required to provide supportive treatment and monitoring. Splenic rupture is a potentially life-threatening complication that occurs in 0.1% to 0.5% of patients with IM.^[14]^ Splenectomy may be needed in cases of splenic rupture, or abscess despite aggressive conservative treatment. In this patient, splenic infarction was successfully treated with supportive therapy without any adverse outcomes. However, hospitalization for close medical monitoring is necessary for most patients with splenic infarction. If there is worsening pain, leukocytosis with persistent fever, or signs of peritonitis, further evaluation is needed to rule out dangerous complications such as splenic abscess or rupture. Therefore, a surgical treatment plan should be in place just in case when the conservative treatment fails.

It is still unknown what period of time is needed for the healing of the infarct tissue, and when it is safe for patients to play sports. Current data on the healing duration of splenic infarction are mostly acquired from the observation of patients who underwent therapeutic splenic infarction via splenic embolization. Lee et al^[15]^ found that the average healing time of splenic infarction confirmed by follow-up CT scan was 43 days in 75 patients. In our case, the patient was concerned about radiation exposure and we did not perform a follow-up CT scan to monitor the recovery of infarction. But an ultrasound examination at 6 weeks after the diagnosis of splenic infarction confirmed the resolution of splenomegaly. We also thought that a repeat CT scan was not so necessary, considering that the patient did not manifest any accompanying symptoms or signs during follow-ups. The patient resumed sports 6 weeks after the diagnosis of splenic infarction and got no any adverse events.

## 5. Limitation

This is a single case report. Considering the nature of the disease, its complicated underlying mechanisms, and potential life-threatening complications, further accumulation of cases is needed to better understand the clinical characteristics and prognosis of splenic infarction in IM. Most importantly, given the lack of prospective data, further studies with more cases are needed to illustrate when, and on what condition, to give the patients recommendations to resume sports

## 6. Conclusion

IM is an acute self-limiting infectious disease caused by EBV. EBV is a prevalent virus that infects more than 90% of the world’s population. Most EBV primary infections occur during childhood and adolescence, and are often asymptomatic; but some patients with EBV infection manifest IM. If a patient with IM develops abdominal pain, splenic infarction should be considered, even without any associated predisposing medical conditions. Contrast-enhanced CT is the imaging modality of choice in suspected cases. Stable splenic infarction can be managed conservatively; however, hospital admission is usually required to provide quality supportive care, close medical monitoring, and thorough evaluation to rule out any potential underlying diseases. A follow-up CT scan maybe needed for reevaluation. Splenectomy is required in cases of progressive infarction with bleeding, abscess, or rupture. IM mostly affects teenagers who are usually physically active, and a common question is when to recommend the resumption of sports. In our case, the patient resumed sports 6 weeks after the diagnosis of splenic infarction without any adverse events. Clinicians must confirm complete resolution of objective symptoms and splenomegaly before giving patient recommendations to resume sports.

## Author contributions

**Writing – original draft:** Lijun Ma.

**Writing – review & editing:** Lijun Ma.
